# Supplementary material for: Association of the KDIGO Risk Classification with the Prevalence of Heart Failure in Patients with Type 2 Diabetes
Source: J Clin Med. 2021 Oct 9;10(20):4634. doi: 10.3390/jcm10204634 (PMC8541098; doi:10.3390/jcm10204634)

Supplementary Table S1. Patients' baseline characteristics based on the type of HF.

VARIABLE		Total group N=1497	Group 1: No heart failure N=943	Group 2: HF with EF < 50% N=360	Group 3: HF with EF ≥ 50% N=194	p	p group 2 vs 3
**Age (years)**		67.3 (10.1)	65.9 (9.9)	68.5 (9.8)	72.3 (9.5)	<0.001	<0.001
**Gender (% female)**		501 (33)	320 (33.9)	76 (21.1)	93 (47.9)	<0.001	<0.001
**Current smoking (%)**		164 (10.8)	116 (12.3)	35 (9.7)	11 (5.7)	<0.001	<0.001
**Hypertension (%)**		1244 (81.9)	763 (80.9)	288 (80)	178 (91.7)	0.001	<0.001
**Chronic obstructive pulmonary disease (%)**		166 (10.9)	79 (8.4)	56 (15.6)	29 (14.9)	<0.001	0.85
**Obstructive sleep apnea (%)**		232 (15.3)	126 (13.4)	65 (18.1)	37 (19.1)	0.03	0.77
**Fatty liver disease (%)**		181 (12.1)	132 (14.2)	26 (7.2)	17 (8.8)	0.001	0.51
**Auricular fibrillation (%)**		327 (21.6)	108 (11.4)	134 (37.2)	83 (42.8)	<0.001	0.20
**Coronary artery disease (%)**		633 (41.9)	339 (36)	219 (60.8)	72 (37.1)	<0.006	<0.001
**Cerebrovascular disease (%)**		128 (8.5)	66 (7)	45 (12.5)	17 (8.8)	0.019	0.18
**Peripheral artery disease (%)**		164 (10.8)	78 (8.3)	57 (15.8)	29 (14.9)	<0.001	0.78
**Atherosclerotic cardiovascular disease (%)**		741 (49)	406 (43)	242 (67.2)	90 (46.4)	<0.001	<0.001
**Number of vascular territories affected**	0	771 (51)	537 (57)	118 (32.8)	104 (53.6)	<0.001	<0.001
	1	577 (38.2)	335 (35.5)	172 (47.8)	67 (34.5)		
	>1	164 (10.8)	71 (7.5)	70 (19.4)	23 (11.9)		
**Charlson index (points)**		0.62 (0.88)	0.52 (0.8)	0.81(1.00)	0.80 (0.98)	<0.001	0.99
**Retinopathy**	**Simple (%)**	114 (7.6)	77 (8.2)	22 (6.1)	10 (5.1)	0.67	0.94
	**Proliferative (%)**	74 (4.9)	45 (4.8)	19 (5.3)	10 (5.1)		
**Body mass index (Kg/m2)**		30.3 (5.2)	30.3 (5.1)	29.6 (5.1)	31.1 (5.9)	0.008	0.01
**Systolic blood pressure (mmHg)**		135 (19.4)	138 (18)	126 (19.6)	135 (19.6)	<0.001	<0.001
**Diastolic blood pressure (mmHg)**		76 (11.5)	77 (11)	72 (11.6)	74 (11.2)	<0.001	0.47
**Diabetes duration (years)**		14 (11.1)	13.7 (9.9)	15.4 (14.4)	13.2 (9.4)	0.023	0.074
**HbA1c (%)**		7.3 (1.3)	7.3 (1.3)	7.2 (1.2)	7.4 (1.4)	0.079	0.30
**Non-HDL cholesterol (mg/dl)**		109 (34.1)	110 (34)	104 (34)	112 (35)	0.005	0.04
**Triglycerides (mg/dl)***		133 (85)	129 (87)	135 (84)	138(75)	0.77	0.99
**Haemoglobin (g/dl)**		14 (1.8)	14.3 (1.7)	13.8 (1.9)	13.2 (1.8)	<0.001	<0.001
**NT-proBNP (pg/ml)***		243 (782)	127 (245)	915(2340)	624 (1660)	<0.001	0.45
**Ejection Fraction (%)**		54.9 (13.4)	61.5 (7.6)	36.6 (9)	60 (7.8)	<0.001	<0.001
**Glomerular filtration rate (ml/min/1,73 m2)**		73.1 (22.7)	77.9 (20.8)	65.4 (23.2)	63.3 (24.3)	<0.001	0.86
**Urinary albumin excretion (mg/g)**		87 (393)	62 (201)	140 (693)	121 (420)	0.009	0.99
**KDIGO risk**	Low (%)	715 (56.4)	518 (63.6)	124 (44.3)	70 (40.9)	<0.001	0.79
	Moderate (%)	295 (23.3)	190 (23.4)	63 (22.5)	42 (24.6)		
	High (%)	152 (12)	70 (8.6)	48 (17.1)	34 (19.9)		
	Very high (%)	106 (8.4)	36 (4.4)	45 (16.1)	25 (14.6)		

Values in the boxes are number of patients (%), mean (standard deviation) or median* (interquartile range).

Supplementary Figure S1. Prevalence of HF type based on patients' concomitant conditions (OSA: obstructive sleep apnoea syndrome; CAD: coronary artery disease; CD: cerebrovascular disease; PAD: peripheral artery disease; ACVD: atherosclerotic cardiovascular disease; AF: atrial fibrillation)

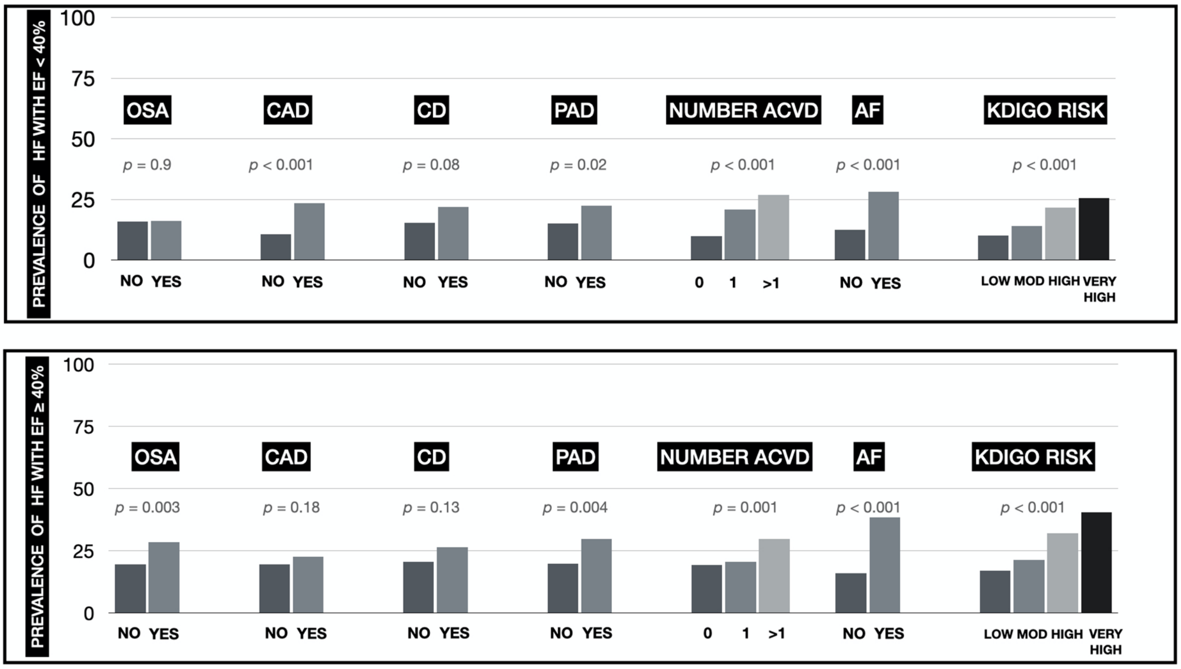

Supplement: Supplementary file 1 [file jcm-10-04634-s001.zip › jcm-1349521-supplementary.pdf]
